# Child-Pugh Score, MELD Score and Glasgow Blatchford Score to Predict the In-Hospital Outcome of Portal Hypertensive Patients Presenting with Upper Gastrointestinal Bleeding: An Experience from Tertiary Healthcare System

**DOI:** 10.3390/jcm11226654

**Published:** 2022-11-09

**Authors:** Zubia Jamil, Shahida Perveen, Samreen Khalid, Mohammed Aljuaid, Memoona Shahzad, Bashir Ahmad, Yasir Waheed

**Affiliations:** 1 Department of Medicine, Foundation University School of Health Sciences, Foundation University Islamabad, DHA Phase I, Islamabad 44000, Pakistan; 2 Department of Health Administration, College of Business Administration, King Saud University, Riyadh 11587, Saudi Arabia; 3 Primary Care, Shoebury Health Center, Campfields Road, Shoeburyness, Southend-on-Sea SS3 9BX, UK; 4 Department of Biotechnology, International Islamic University, Islamabad 44000, Pakistan; 5 Office of Research, Innovation & Commercialization, Shaheed Zulfiqar Ali Bhutto Medical University (SZABMU), Islamabad 44000, Pakistan; 6 Gilbert and Rose-Marie Chagoury School of Medicine, Lebanese American University, Byblos 1401, Lebanon

**Keywords:** upper gastrointestinal bleeding, endoscopy, portal hypertension

## Abstract

The two most familiar scores used for prognostication of liver cirrhosis are the Model for End-stage Liver Disease (MELD) and Child-Turcotte-Pugh (CTP), while the Glasgow-Blatchford (GB) score is used for sorting non-variceal upper gastrointestinal hemorrhage into high- or low-risk categories. This study evaluates the validity of the CTP, MELD, and GB scoring systems in prognosticating the in-hospital outcome of bleeding portal hypertensive patients. In this study, the ROC curve and Younden index determine the efficacy of three scoring systems. The results indicate that CTP was the most efficient score as the predictor of outcome (AUC = 0.9, cut-off value > 7); followed by MELD (AUC = 0.8, cut-off value > 18) and then the GB score (AUC = 0.64, cut-off value > 14) (*p* < 0.05). In pair-wise comparison, the difference between CTP and MELD was insignificant (*p* > 0.05). Patients with a CTP score of >7 had notably higher in-hospital mortality (19.8% vs. 0.9%, *p* < 0.0001). Similarly, mortality with a MELD score > 18 was significant (14.8% vs. 5.9% (*p* < 0.0001). The GB score was not a good indicator of the outcome. Platelets, albumin, CTP, and MELD scores were the independent contributors to mortality. Thus, as liver cirrhosis prognosticators, CTP and MELD scores can also both be used as predictive scores of the in-hospital outcomes of bleeding patients due to portal hypertension. Compared to the GB score, CTP and MELD scores are fairly efficient predictors in these patients.

## 1. Introduction

Acute bleeding in the upper gastrointestinal region is one of the most encountered medical emergencies [1] Common causes are variceal hemorrhage and peptic ulcers due to *H. pylori* or NSAID use [2,3]. Endoscopy allows for the identification of the source of bleeding as well as hemostatic treatment of actively bleeding lesions [3]. Despite recent advancements in the management techniques, the mortality rate related to upper gastrointestinal bleeding remains persistently high and accounts for about 10–14% of all deaths related to bleeding from any part of gastrointestinal tract [4,5]. Therefore, sorting early risk with respect to different clinical assessment scores in patients presenting with GI bleed in the upper tract can improve survival in these patients.

Several prognostic scoring systems have been established to identify high-risk patients who need urgent treatment for upper GI bleeding and low-risk patients for whom endoscopy can be postponed [6]. To determine the prognosis of liver cirrhosis in patients, there are two frequently used scores; one is Child-Turcotte-Pugh(CTP) and the other one is Model for End-stage Liver Disease (MELD) [7]. Alternatively, there is another score known as the Glasgow Blatchford score that is used to classify patients experiencing upper gastrointestinal hemorrhage into high- or low-risk categories to predict the early need for interventions and clinical outcomes [8].

CTP score is a widely credited scoring system that is useful in predicting the survival of liver cirrhosis patients, assessing bleeding and encephalopathy [9]. Similarly MELD score was categorically created to help predict three-month survival in patients having liver cirrhosis [10]. Both of these scores are extensively acknowledged to apprehend variceal bleeding in portal hypertensive patients. Some studies consider CTP score better than MELD score, while others favor the superiority of MELD in predicting clinical outcomes of bleeding esophageal varices [9].

The Glasgow-Blatchford scoring system comprises clinical and laboratory parameters to classify patients experiencing upper gastrointestinal bleeding into high- or low-risk categories for management purposes (endoscopic ± surgical intervention vs. medical management alone) [8]. It has been widely used in both cirrhotic and non-cirrhotic patients. However, it is more accurate to predict composite outcomes, including the duration of hospital stay, intervention and mortality in patients experiencing non-portal hypertensive bleeding rather than portal hypertensive bleeding [11]. In this study, the Glasgow-Blatchford scoring system is used in comparison with the Child-Pugh score and MELD score to evaluate its any effectiveness in portal hypertension bleed.

Considering the guarded prognosis of patients presenting with upper gastrointestinal bleeding, it is pertinent to use appropriate scoring systems capable of early risk stratification. This will allow for effective triage and an early escalation for appropriate treatment in high-risk patients, thus achieving lower mortality and health maintenance expenditure [12].

The main purpose of the study was to evaluate the clinical application of cirrhosis prognosis scores such as the CTP, MELD, and Glasgow Blatchford (GB) scores in foreseeing the in-hospital outcome of portal hypertensive patients experiencing bleeding in the upper gastrointestinal region.

## 2. Materials and Methods

Prospective analytical study was conducted at Fauji Foundation Hospital, Rawalpindi, from October 2021 to April 2022. Consent was obtained from the ethical panel of the hospital as per rules on 23 October 2021 (Ref No. 324/RC/FFH/RWP).

### 2.1. Study Cohort

Patients were educated about the experimental design and purpose before enrolling. In the case of unstable patients, the consent of first-degree relatives was taken, and an ethical committee was informed about this proxy consent.

### 2.2. Criteria for Inclusion of Patients

All of the patients presenting with bleeding in the upper gastrointestinal tract (coffee-ground vomiting, hematemesis, melena, or hematochezia) at the mentioned time were included in the study cohort provided the cause of bleeding was portal hypertension.

Portal hypertension was diagnosed if:

1. The patient had a preceding history of liver cirrhosis leading to portal hypertension (evident by hospital records, laboratory and radiological investigations, or endoscopic findings);
2. Any existing risk factor that can lead to liver cirrhosis and portal hypertension was evident by one or more than one of the following parameters:
  a. The diameter of Portal vein was greater than 15 mm on ultrasound along with coarse liver texture.
  b. Platelet count <100,000/mcL; as thrombocytopenia is an indirect marker of portal hypertension [13].
  c. Upper GI endoscopy showed evidence of portal hypertension (esophageal or fundal varices ± portal hypertensive gastropathy).

### 2.3. Criteria for Exclusion of Patients

The following criteria were used to exclude the patients.

1. The patients with incomplete or missing laboratory results because biochemical parameters were required for calculating various scores tested in this study.
2. Patients who did not give consent.
3. Patients who did not have evidence of portal hypertension or in whom cause of bleeding was found to be non-portal hypertensive after upper GI endoscopy.

### 2.4. Methodology

A detail of the patient’s personal profile, including name, age, address, hospital registration number and personal phone number was taken at the time of hospital admission. History focusing on the time of onset of bleeding and its possible etiological factors (presence of co-morbidities, medication, lifestyle and dietary history) was taken by post-graduate trainees or senior registrar. Previous medical records were also analyzed simultaneously to determine the reason for bleeding in the gastrointestinal region in these patients.

After detailed examination of patients, laboratory tests were taken by expert phlebotomist. Complete blood picture, coagulation profile (INR, PT), tests for renal and liver function were sent at the time of admission. All of the patients were started on standard treatment for variceal bleeding from the time of admission, which included prophylactic antibiotics (third generation cephalosporins 1 g/day for 5–7 days), terlipressin, proton pump inhibitors, transfusion of blood products.

Each patient underwent upper gastrointestinal endoscopy to diagnose the reason for bleeding. Sedation and sterilization were carried out according to standard measures. One endoscopist performed endoscopy to lessen the intra-observational and inter-observational variations (endoscope XP180; Olympus Company, Tokyo, Japan).

After endoscopy, those patients in whom the cause of bleeding was due to portal hypertension (esophageal or fundal varices) were added to the study, while the other patients who were bleeding because of other causes were excluded because all three scores, the CTP score, the MELD score, and the GB score, can be used for the portal hypertensive group only.

Three scoring systems were calculated for each portal hypertensive patient.

#### 2.4.1. Child-Turcotte-Pugh (CTP) Score

Five variables are used to calculate the CTP score (ascites, hepatic encephalopathy, INR, bilirubin, and albumin). Each variable was scored from 1 to 3 depending on the condition of the patient and laboratory result [14].

#### 2.4.2. MELD Score

A standard formula is used to calculate MELD scores [10].

MELD = 3.78 × log [serum bilirubin (mg/dL)] + 11.2 × lo [INR] + 9.57 × log [serum creatinine] + 6.43

#### 2.4.3. Glasgow Blatchford (GB)Score

The GB score was calculated using nine variables (hemoglobin, serum urea level, systolic BP and heart rate at time of presentation, gender of patient, history of melena and syncope, history of hepatic disease along with cardiac failure). The score varied between 0 to 23 [15].

The MDCalC calculator was used to calculate these three scores of patients.

The individual identity of patients was concealed throughout the data collection and analysis to maintain the patient’s privacy. The prime endpoint of the study was the outcome of patients whether the patient survived and was discharged from the hospital or patients died.

### 2.5. Statistical Analysis

Data were added and analyzed using MedCalc Statistical Software 19.6.4 (MedCalc Software, Ostend, Belgium). Mean (ranges and standard deviation) and Median (IQR) were used for quantitative parameters, while percentages were used for qualitative parameters. The quantitative variables among the two categories were examined by the Student’s *t*-test while the Chi-Square test was implied to evaluate the qualitative variables. MDCalc calculator was used to calculate three scores (MELD, CTP, and GB scores) of portal hypertensive patients. The Receiver Operating Characteristic Curve (ROC Curve) was applied to determine the usefulness of three of these scores in determining the end result of portal hypertensive patients with upper gastrointestinal bleeding. The cutoff value for each score in relation to sensitivity, specificity, negative and positive probability ratios along with 95% confidence interval as well as *p*-value was also calculated by the Youden index. The survival duration was taken from the time of admission to the hospital until the outcome and expressed in hours. Log Rank along with Kaplan-Meier analysis was used to analyze the percentages of survival and duration of hospital stay of portal hypertensive patients in relation to these three scores.In the end, Cox regression analysis was used to analyze single determinants affecting the survival of the study cohort. The Omnibus test was used to test the model efficacy and all determinants were expressed as odd ratio in relation to *p*-value and 95% confidence interval.

## 3. Results

A total of 259 patients presented with bleeding in the upper gastrointestinal tract during the given period. Among 259 patients, 83.8% (*n* = 217) had upper gastrointestinal (GI) bleeding due to portal hypertension, while 16.2% (*n* = 42) had non-portal hypertensive causes of upper GI bleeding, thus excluding them from the study.

The mean age of the experimental group was 60.71 ± 14.07 (16–92) years; 93.5% (*n* = 203) were females and 6.5% (*n* = 14) were males. The biochemical features of the study cohort at the time of admission are shown in Table 1.

Portal hypertension, secondary to Hepatitis C-related cirrhosis, accounted for 89.9% (*n* = 195) patients with an upper GI bleed. Among these patients, 53.8% (*n* = 105) did not receive any treatment for Hepatitis C infection, 28.2% (*n* = 55) took interferons for Hepatitis C infection, 10.3% (*n* = 20) were treated with interferons initially and then direct acting antivirals and only 7.7% (*n* = 15) were treated with Direct Acting Antivirals for Hepatitis C infection. Only a trivial portion of patients, 4.1% (*n* = 8), had achieved a sustained viral response.

Non-cirrhotic portal hypertension resulted in 6.5% (*n* = 14) patients while 3.6% (*n* = 8) patients had portal hypertension complicated by hepatocellular carcinoma.

Diabetes Mellitus was the main comorbid condition affecting 48.8% (*n* = 106) patients, 43.8% (*n* = 95) had hypertension, 36.9% (*n* = 80) had a history of ischemic heart disease, 20.7% (*n* = 45) had chronic obstructive pulmonary disease and 4.6% (*n* = 10) had a previous history of cerebrovascular accident.

At the time of admission, 97.7% (*n*= 212) patients were hemodynamically stable and 2.3% (*n* = 5) were unstable, they were shifted to medical ICU. Among unstable patients, three died and two survived. The mean systolic blood pressure of patients was (132.89 ± 24.15) (range: 85–180) mmHg and diastolic blood pressure was (93.88 ± 12.05) (range: 50–100) mmHg. Mean pulse was (99.23 ± 14.23) (range: 60–150) bpm and mean temperature was (98.89 ± 9.50) (range: 98.5–100) ℉.

The main goal of the experimental design was to assess the clinical application of MELD, CTP, and GB scores in determining the in-hospital outcome of portal hypertensive patients presenting with upper gastrointestinal bleed.

### 3.1. ROC Curve of CTP, MELD and GB Scores

The main endpoint of the study was the in-hospital outcome (death or survival) of portal hypertensive patients. Among 217 portal hypertensive patients, 79.3% (*n* = 172) patients survived while 20.7% (*n* = 45) died. Among the 45 patients who died, 26.7% patients (*n* = 12) died from hypovolemia secondary to gastrointestinal hemorrhage, 24.4% patients (*n* = 11) due to cardiac cause, 24.4% (*n* = 11) due to respiratory failure, 13.3% (*n* = 6) due to terminal malignancy and only 11.1% (*n* = 5) from septicemia.

The mean CTP score was 8.12 ± 2.18 (5–15), the MELD score was 13.65 ± 6.48 (6–35) and the GB score was 10.54 ± 3.83 (1–19).

AUC—area under the curve calculated by ROC was used to establish the efficacy of the CTP score, the MELD score, and the GB score in determining the in-hospital survival of portal hypertensive patients who had upper gastrointestinal bleeding.

The AUC for CTP score was the highest (AUC = 0.9, SE = 0.03, 95% CI = 0.82–0.90) and that for MELD score was (AUC = 0.8, SE = 0.03, 95% CI = 0.76–0.87). The GB score was not found to be a good scoring system in predicting the in-hospital survival of these patients (AUC = 0.64, SE = 0.04, 95% CI = 0.58–0.71). To determine the cut-off value of each scoring system with its sensitivity, specificity, positive, and negative likelihood ratios, Youden index is used. The AUC, cut-off value with its sensitivity, specificity, positive and negative likelihood ratios with 95% confidence interval and *p*-value of three scoring systems in determining in-hospital survival of these patients are shown in Table 2.

**Figure 1:**
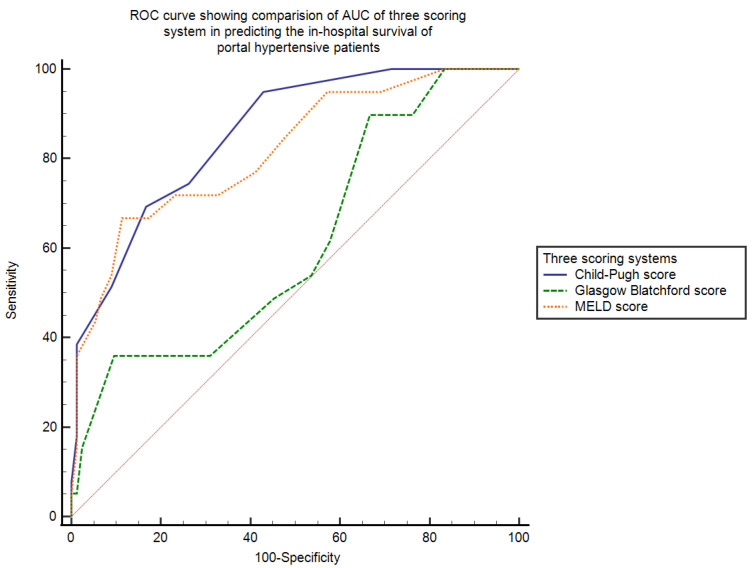
This figure shows comparison of AUC of three scoring systems (CTP, MELD, and GB scores) in predicting the outcome of bleeding portal hypertensive patients. AUC is highest for the CTP score (0.9); the MELD AUC = 0.8 while lowest for the GB score = 0.64. CTP: Child-Turcotte-Pugh, MELD: Model of end stage liver disease, GB score: Glasgow Blatchford score.

When we pairwise compared the AUC of the three scoring systems, we found that the difference between AUCs of CTP~MELD was just 0.04 (SE = 0.03, 95% CI = −0.03–0.10, *p* = 0.24), theCTP~GB score was 0.25 (SE = 0.05, 95% CI = 0.15–0.34, *p* < 0.0001) and the MELD~GB score was 0.20 (SE = 0.05, 95% CI = 0.09–0.31, *p* = 0.0002). This showed that both MELD and CTP scores are significantly better than the GB score in determining the in-hospital survival of portal hypertensive patients who had upper gastrointestinal bleeding. The ROC curve showing the AUC of the three scoring systems, the Child–Pugh score, the MELD score, and the Glasgow Blatchford score, in calculating the in-hospital survival of portal hypertensive patients presenting with bleeding in the upper gastrointestinal tract is shown in Figure 1.

**Figure 2:**
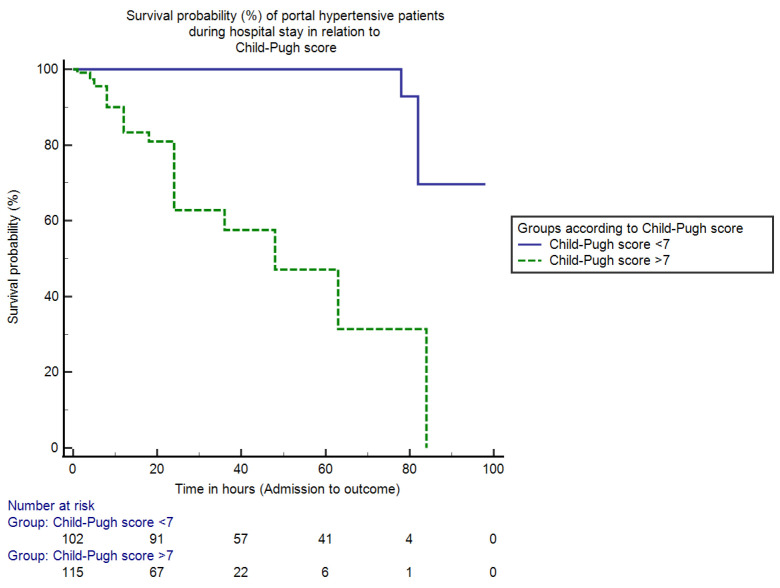
This is a figure showing survival percentages (%) of portal hypertensive patients during the hospital stay in relation to the Child-Pugh score. The mortality (in-hospital) for hospitalized persons with CTP score ≤ 7 was only 0.9% and for patients with CTP score > 7 was 19.8% (*p* < 0.0001). Moreover, the duration (median) of hospital stay for patients with CTP score ≤ 7 and >7 was 92 h and 48 h respectively (*p* < 0.0001).

The mean duration of in-hospital stay from admission to the outcome was 35.02 ± 21.24 (2–98) h. Patients were divided into two groups according to the cut-off value of CTP score calculated using the Youden index: patients with a CTP score < 7 and patients with a CTP score > 7. The in-hospital mortality for patients with a CTP score < 7 was only 0.9% (2/217) and the mortality percentage for patients with a CTP score > 7 was 19.8% (43/217) (95% CI = 13.59–24.74, *p* < 0.0001). The median duration of hospital stay for the admitted cohort with a CTP score < 7 was 92 h (IQR = 4, HR = 0.07, 95% CI = 81.19–99.52), whereas for patients with a CTP score > 7 it was 48 h (IQR = 8, HR = 12.64, 95% CI = 41.88–56.87, *p* < 0.0001). The Kaplan–Meier analysis curve that shows the overall in-hospital survival probability in the two groups of portal hypertensive patients presenting with upper gastrointestinal bleeding in relation to the Child–Pugh score is shown in Figure 2.

**Figure 3:**
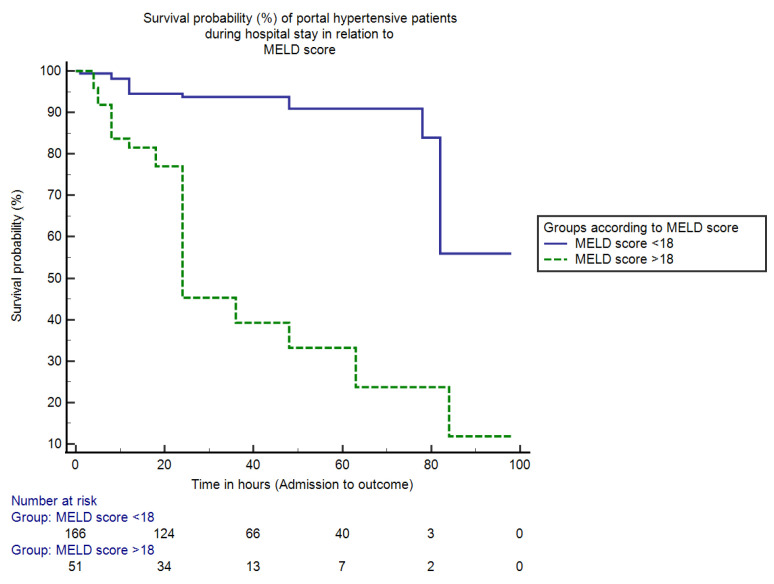
This is a figure showing survival percentages (%) of portal hypertensive patients during the hospital stay in relation to the score of MELD. The mortality (in-hospital) for hospitalized persons with MELD score ≤ 18 was only 5.9% and for patients with MELD score > 18 was 14.8% (*p* < 0.05). The duration (median) of hospital stay for patients with MELD score ≤ 18 and >18 was 85 h and 24 h respectively (*p* < 0.0001).

Similarly, patients were categorized according to the MELD score cut-off value.The in-hospital mortality for patients with MELD score < 18 was 5.9% (13/217) and the mortality percentage for patients with MELD score > 18 was 14.8% (32/217) (95% CI = 3.19–14.76, *p* < 0.05).The duration (median) of hospital stay for hospitalized persons with MELD score < 18 was 85 h (IQR = 4, HR = 0.04, 95% CI = 77.08–93.52), whereas for patients with a MELD score > 18 it was only 24 h (IQR = 5, HR = 21.35, 95% CI = 32.48–52.19, *p* < 0.0001). The Kaplan–Meier analysis curve that shows the overall in-hospital survival probability in two groups of portal hypertensive patients having bleeding in the upper gastrointestinal tract in relation to MELD score is shown in Figure 3.

**Figure 4:**
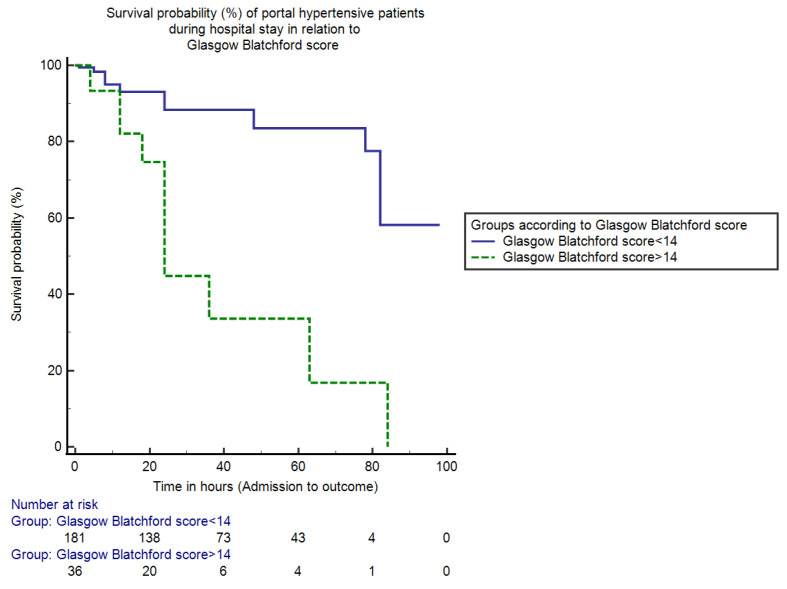
This is a figure showing survival percentages (%) of portal hypertensive patients during the hospital stay in relation to the GB score. The mortality (in-hospital) for hospitalized persons with GB score ≤ 14 was only 11.5% and for patients with GB score > 14 was 9.2% (*p* > 0.05). The duration (median) of hospital stay for hospitalized persons with GB score ≤ 14 and >14 was 81 h and 38 h respectively (*p* < 0.0001).

In the end, patients were grouped into two categories according to the cut-off value of the GB score.The in-hospital mortality for patients with GB score < 14 was 11.5% (25/217) and the mortality percentage for patients with GB score > 14 was 9.2% (20/217) (95% CI = −3.52–8.16, *p* > 0.05).The (median) duration of hospital stay for patients with a GB score < 14 was 81 h (IQR = 6, HR = 0.05, 95% CI = 74.54–88.58), whereas for patients with a GB score > 14 it was 38 h (IQR = 5, HR = 19.70, 95% CI = 27.88–49.82, *p* < 0.0001). The Kaplan–Meier analysis curve showing overall in-hospital survival probability in two groups of portal hypertensive patients presenting with upper gastrointestinal bleeding in relation to Glasgow Blatchford score is shown in Figure 4.

### 3.2. Survival in Relation to Single Determinant

To find factors affecting the outcome of portal hypertensive patients presenting with gastrointestinal bleeding, all variables were analyzed by linear regression analysis first, and then statistically significant factors were further scrutinized by Cox regression analysis. Omnibus tests showed that the model was statistically significant (Chi-Square test = 59.12, *p* = 0.00). Platelet count, serum albumin, CTP, and MELD scores were found to be predictors of survival in portal hypertensive patients (*p* < 0.05); the Glasgow Blatchford score did not affect the clinical outcome in these patients. The various predictors of survival among the study cohort are shown in Table 3.

## 4. Discussion

The clinical scoring systems (CTP, MELD, and GB) for patients with portal hypertension and bleeding in the upper gastrointestinal tract were developed to differentiate high-risk groups from low-risk groups, guide their prompt management and thus significantly contribute to reducing mortality in this cohort [12,16]. The utilization of such scoring systems with appropriate clinical assessment helps in identifying individuals who are at low risk of complications for outpatient endoscopy and early discharge [17].

Since the evaluation of the three scoring systems (MELD, Child-Pugh score and GB score) to anticipate the survival outcomes was applicable only to patients with gastrointestinal bleeding secondary portal hypertension, we studied only the portal hypertensive group that constituted 83.8% (*n* = 217) of the study cohort. Our study demonstrated that Child-Pugh-Turcotte and MELD scores are excellent predictors of survival probability in these patients, while the Glasgow Blatchford score did not prove accurate in determining the in-hospital survival. Similar to our results, Peng Y, et al. [18] also showed MELD and CTP scores to be accurate forecasters of risk assessment in upper gastrointestinal bleeding due to liver cirrhosis.

In the study group, the mortality rate among bleeding portal hypertensive patients was 20.7%, while 79.3% survived. These results are closer to a multicenter analysis by Fortune, B.E., et al. [19] where the mortality rate was observed around 25% in patients with variceal hemorrhage. Another study reported a high mortality of around 30% in such patients, signifying a high disease burden [20].

The efficacy of each score was determined by area under the curve calculated by ROC, which came out to be highest for the CTP score (AUROC = 0.9) and the MELD score (AUROC = 0.8), thus proving them to be excellent scoring systems for predicting the survival outcomes in bleeding portal hypertensive patients. Comparable results were observed by Peng, Y., et al. [18] in which AUROCs for CTP and MELD score were nearly 0.8 when assessing the survival probability of patients with cirrhosis and upper GI bleed. Similarly, a study by Hsu, S.C., et al. [21] who compared three scoring systems (MELD, GB, and Rockall score) to predict mortality in bleeding cirrhotic patients found that MELD is superior to the other two scores in assessing survival outcomes of these patients.

However, the GB score had the lowest AUROC in our study (AUC = 0.6) and did not prove efficient in predicting survival outcomes in these patients. Similarly, in favor of our results, the GB score was not precise in assessing survival probability in patients with bleeding varices in another clinical study by Thanapirom, K., et al. [22].

While comparing the scores pairwise through AUC (area under the curve), a minimum difference between AUCs of CTP versus MELD scores were observed, while it was higher upon comparing GB score with MELD score and CTP scores, proving that both MELD and CTP scores are superior to the Glasgow Blatchford score for estimating the survival outcomes in these patients. Similar to our findings, Tantai, X., et al. [23] proved that upon comparisons of AUCs, CTP and MELD scores both proved efficient in predicting in-hospital mortality, while the GB score had a small AUC depicting low predictive ability in survival outcomes.

In the Child–Pugh scoring system, patients with a CTP score < 7 had a median duration of stay of 92 h with the lowest mortality of 0.9% compared to CTP > 7 (48 h and 19.8%) (*p* < 0.05). This shows that the patients with score < 7 have greater survival duration and lesser mortality percentages compared with patients with CTP > 7. Corresponding to our results, the overall survival decreases as the CTP score progresses, which is evident in some studies, and the mortality rate was reported to be 3 times higher with a CTP > 10.5 [24,25].

Similarly, by applying the MELD scoring system, hospitalized persons with a MELD score < 18 had a duration (median) of hospital stay of 85 h and mortality of 5.9% vs. MELD score > 18 (24 h and 14.8%) (*p* < 0.05). This shows that the patients with a score <18 have greater survival duration and lesser mortality percentages compared to patients with MELD > 18. Our findings are comparable with the clinical study by Reverter, E., et al. [26] in terms of MELD score, who found its cutoff value of 19 for mortality prediction and showing that a score > 19 had a mortality of around 20%, whereas a score < 11 had a mortality of about 5%. Both scores (MELD and CTP) have similar results, in which lesser scores have prolonged the survival duration and decreased the mortality rate.

In the case of the Glasgow Blatchford scoring system, patients with low scores < 14 had a longer duration of stay but with a higher mortality rate compared to patients with high GB scores (81 h vs. 38 h; 11.5% vs. 9.2%, *p* > 0.05) proving this score to be an unreliable indicator for assessing the survival probability of portal hypertensive patients with upper GI bleed, shown by another study of Thanapirom, K., et al. as well [22]. Although a lesser score is associated with longer hospital stay, it is also associated with higher mortality percentages, proving this score to be an unconvincing score for these patients.

Low platelet count, hypoalbuminemia, high MELD, and CTP scores in portal hypertensive hospitalized persons with bleeding from the upper gastrointestinal tract were found to be statistically significant independent prognostic markers of survival outcomes, while GB score was not found to be an accurate prognosticator in these patients using regression analysis. In a case study by Fortune, B.E., et al. [19] CTP and MELD scores proved to be self-determining forecasters of in-hospital outcomes, while the CTP score ranked highest in predicting mortality and stratifying the risk.

The key limitation of this experimental study was that it compared the potency of these three scores for determining the outcome only during the in-hospital stay. Further studies are required to evaluate the effectiveness of these scoring systems for longer duration, such as 3 to 6 months with outpatient follow-ups etc. and also involving a greater number of patients and multi-centered approach.

## 5. Conclusions

To conclude, upper gastrointestinal bleeding requires rapid assessment and early intervention to reduce mortality as it is a potentially life-threatening condition. Child-Pugh and MELD scoring systems are effective prognostic tools in assessing survival outcomes of portal hypertensive patients with bleeding from the upper gastrointestinal tract. However, Glasgow Blatchford scores did not prove efficient in predicting the survival probability in this cohort of portal hypertensive patients.

## Figures and Tables

**Table 1:** This is a table demonstrating the baseline characteristics of 259 patients in study group. Standard deviation, Mean and range are used for expression of variables.

Variables	Minimum	Maximum	Mean ± SD
Age (years)	16	92	60.65 ± 14.09
Hemoglobin (g/dL)	4.80	16.50	8.98 ± 2.99
WCC × 10^3^ cells/L	1.19	30.00	8.44 ± 5.05
Platelets × 10^3^ cells/L	15.00	687.00	148.06 ± 105.52
Bilirubin (µmol/L)	3.00	265.00	24.78 ± 32.23
AST (IU/L)	15.00	244.00	53.79 ± 34.29
ALP (IU/L)	55.00	1102.00	205.28 ± 140.28
PT (s)	1.00	40.00	4.75 ± 2.20
INR	1.00	7.30	1.35 ± 0.75
Albumin (g/L)	1.5	6.0	2.90 ± 0.71
Urea (mmol/L)	3.00	42.30	11.22 ± 8.25
Creatinine (μmol/L)	4.20	675.00	128.51 ± 107.44

**WCC:** White cell count, **AST:** Aspartate Aminotransferase, **INR:** International normalized ratio, **PT:** Prothrombin time, **ALP:** Alkaline phosphatase.

**Table 2:** This is a table showing the AUC, cutoff value with its specificity, sensitivity, positive (+) and negative (−) probability ratios with 95% confidence interval and *p* value of three scoring systems in determining in-hospital survival of portal hypertensive patients presenting with upper gastrointestinal bleeding.

Variables	AUC	Cutoff Value	Sensitivity	95% CI	Specificity	95% CI	+LR	−LR	*p*-Value
CTP	0.9	>7	94.9%	82.7–99.4	58.1%	50.4–65.6	2.27	0.08	<0.0001
MELD	0.8	>18	66.7%	49.8–80.9	88.9%	83.3–93.2	6.04	0.37	<0.0001
GB	0.6	>14	41.9%	27.0–57.9	90.5%	85.0–94.5	4.40	0.64	0.002

**AUC:** Area under the curve, **CI:** Confidence Interval, **MELD:** Model of end stage liver disease, **+LR:** Positive Likelihood ratio, −**LR:** Negative Likelihood ratio, **CTP score:** Child-Pugh-Turcotte score, **GB score:** Glasgow Blatchford score.

**Table 3:** This is a Table showing various variables according to cox regression analysis as predictors of survival of bleeding portal hypertensive patients.

Variables	OR (95% CI)	*p* Value
Platelets × 10^3^ cells/L	0.99 (0.98–1.00)	0.01
Albumin (g/L)	0.87 (0.82–0.93)	0.01
CTP score	1.64 (1.27–2.11)	0.00
MELD score	1.12 (1.03–1.21)	0.01
GB score	1.04 (0.92–1.17)	0.51

**MELD:** Model of End-Stage Liver Disease, **GB score:** Glasgow Blatchford score, **CTP score:** Child-Turcotte-Pugh score. Statistically significant results are shown in bold.

## Data Availability

Not applicable.
